# Supplementary figures and images for: Adaptation of the late ISC pathway in the anaerobic mitochondrial organelles of Giardia intestinalis
Source: PLoS Pathog. 2023 Oct 4;19(10):e1010773. doi: 10.1371/journal.ppat.1010773 (PMC10578589; doi:10.1371/journal.ppat.1010773)

Supplementary Figure 1

A

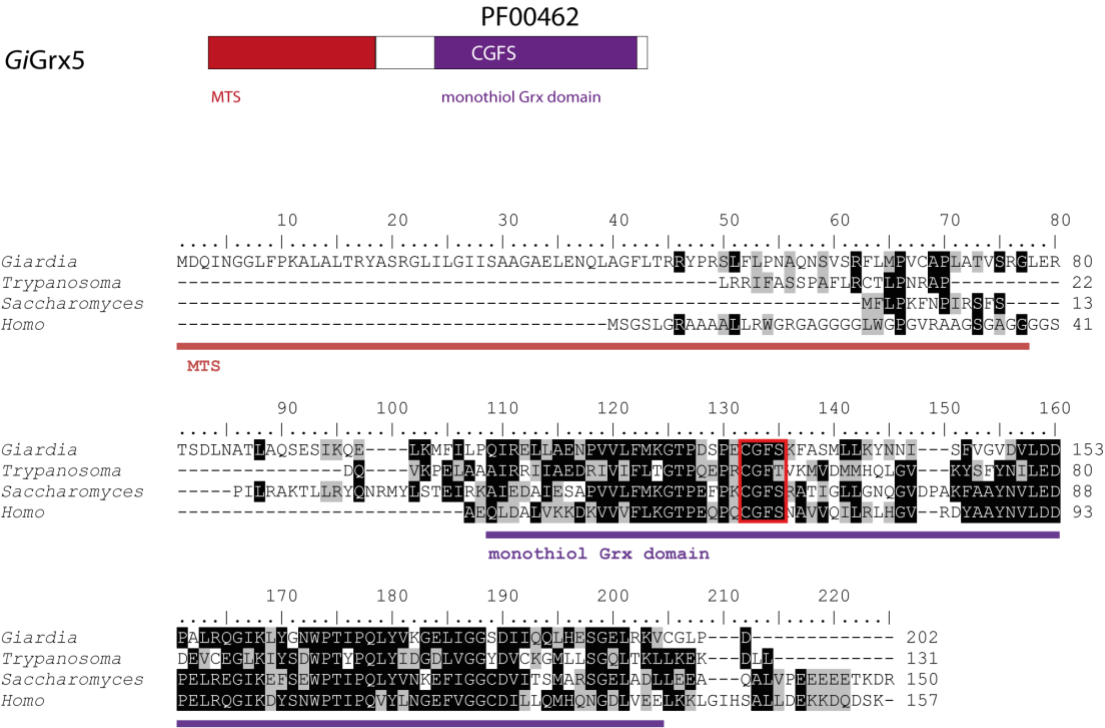

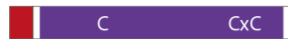

GiNfu1

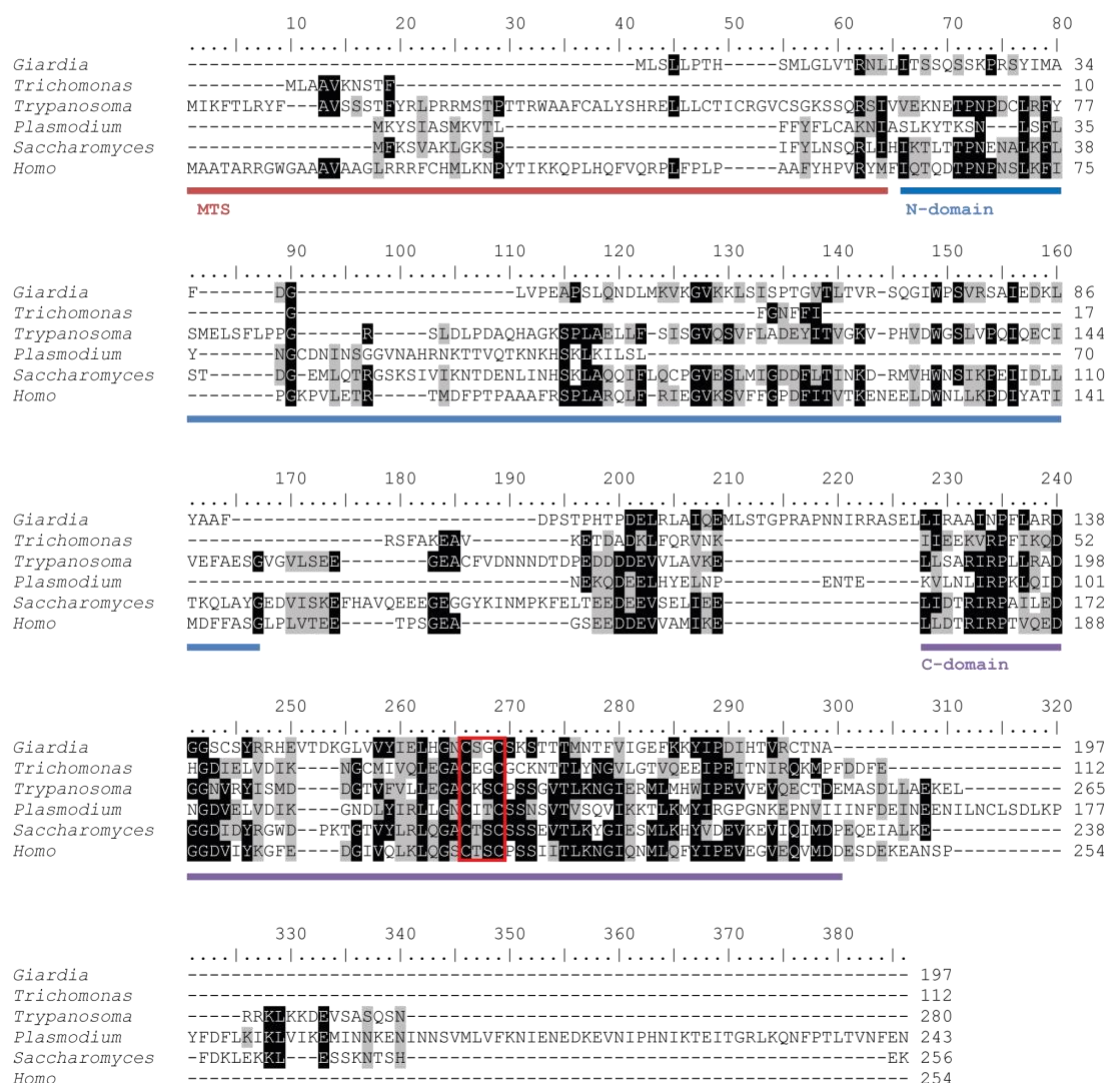

Supplement: S1 Fig — (A) Grx5, the diagram shows the domain structure of GiGrx5, mitochondrial targeting sequence (MTS) is shown in red, monothiol glutaredoxin domain (PF00462) in purple, the CGFS motif is also highlighted. (B) GiIscA2 shares the Fe-S_biosyn domain (PF01521) with the conserved cysteine residues involved in cluster binding. (C) GiNfu1 contains conserved N- and C- domains, the latter of is recognized as NifU domain (PF01106) and carries conserved cysteine motif. (PDF) [file ppat.1010773.s001.pdf]

### Supplementary Figure 3

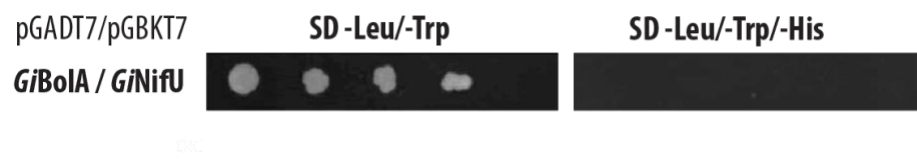

Supplement: S3 Fig — (PDF) [file ppat.1010773.s003.pdf]

## Supplementary Figure 4

$\Delta$ bolA

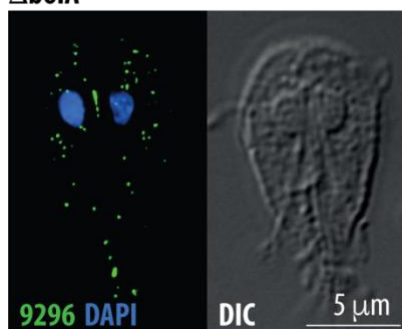

control

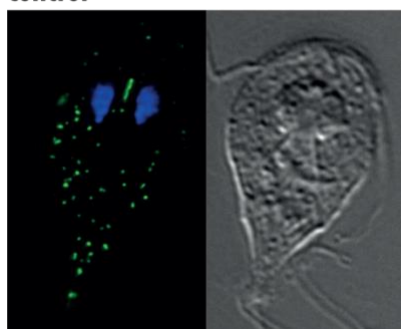

Supplement: S4 Fig — The exemplary image of mitosomes visualized by immunofluorescence microscopy in the ΔbolA1 and control (Cas9) cell lines. Mitosomes were detected by rabbit polyclonal antibody raised against GL50803_9296, the nuclei were stained with DAPI. (PDF) [file ppat.1010773.s004.pdf]

Supplementary Figure 5

A

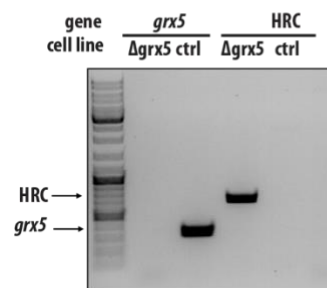

B

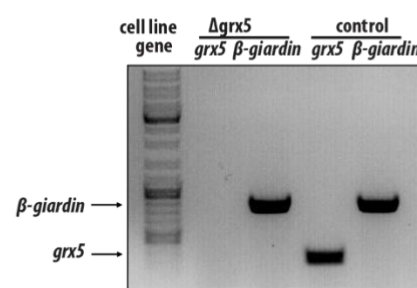

C

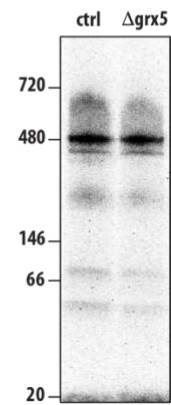

Supplement: S5 Fig — The Δgrx5 cell line was tested for the presence of grx5 gene and the integration of homologous recombination cassette (HRC) by PCR on gDNA, (B) the expression of grx5 gene in Δgrx5 cell line was tested by PCR on the cDNA, β-giardin was used as a control gene, (C) Incorporation of 55Fe to G. intestinalis proteins after 72 h incubation with radioactive iron isotope in the form of ferric citrate. Comparisons of control and Δgrx5 cell extracts show comparable levels of iron incorporation. (PDF) [file ppat.1010773.s005.pdf]
