# Supplementary material for: Adaptation of the late ISC pathway in the anaerobic mitochondrial organelles of Giardia intestinalis
Source: PLoS Pathog. 2023 Oct 4;19(10):e1010773. doi: 10.1371/journal.ppat.1010773 (PMC10578589; doi:10.1371/journal.ppat.1010773)

Supplementary Figure 2

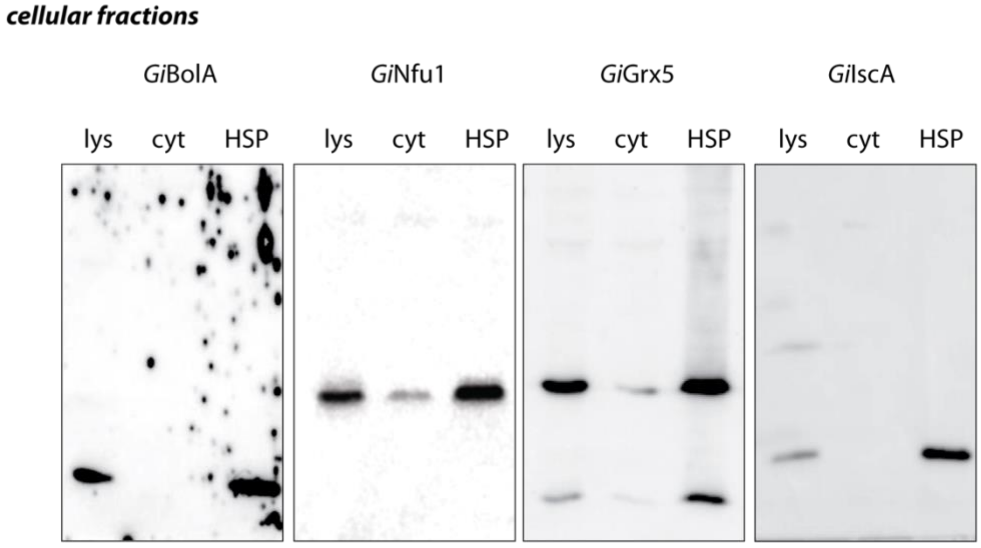

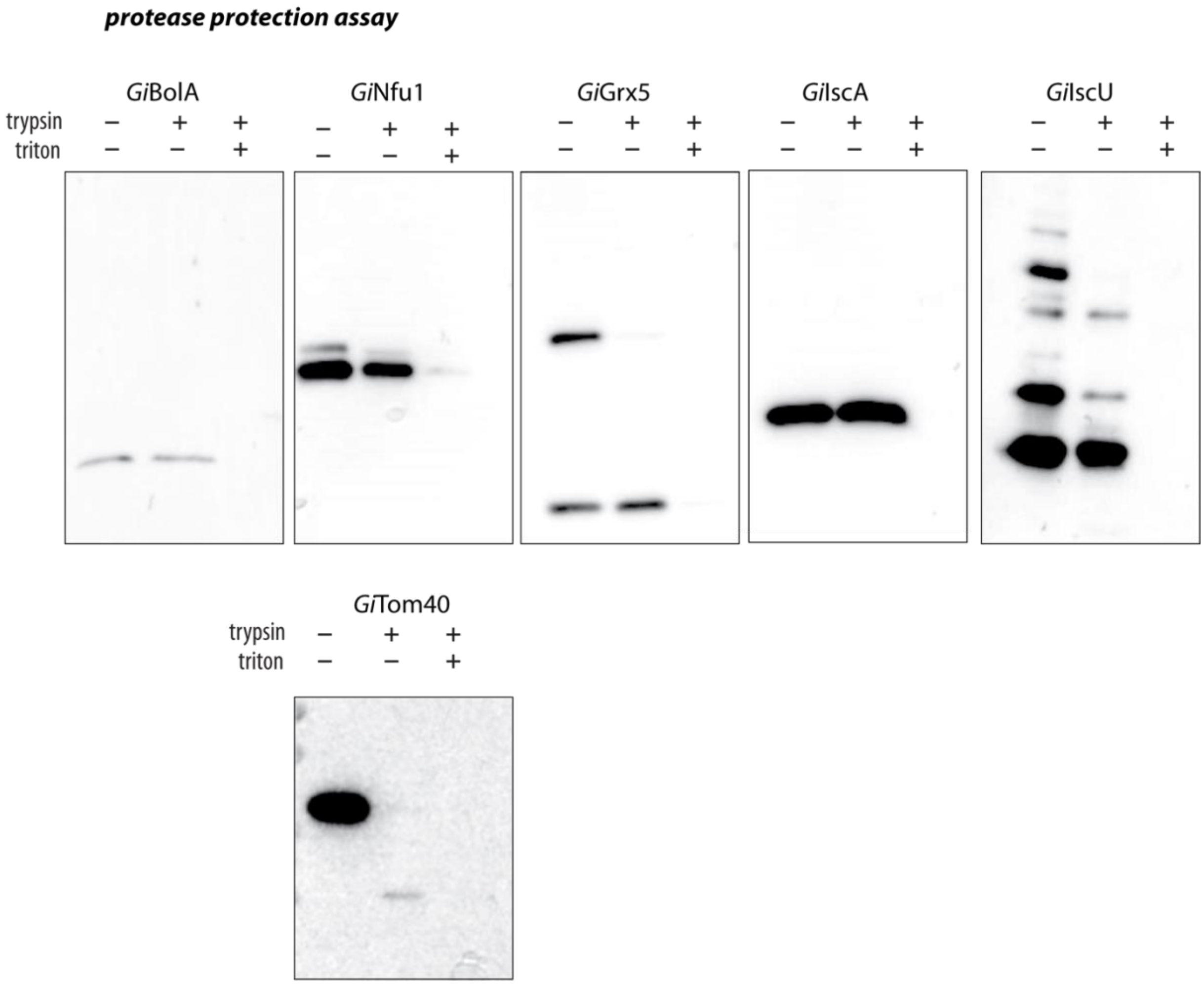

Supplement: S2 Fig — (PDF) [file ppat.1010773.s002.pdf]
