# Supplementary material for: Adaptation of the late ISC pathway in the anaerobic mitochondrial organelles of Giardia intestinalis
Source: PLoS Pathog. 2023 Oct 4;19(10):e1010773. doi: 10.1371/journal.ppat.1010773 (PMC10578589; doi:10.1371/journal.ppat.1010773)
Supplement: Attachment — Submitted filename: Comments for Authors 220823.pdf [file ppat.1010773.s011.pdf]

**Comments for Authors**

Mitosome in *G. intestinalis* is one of the simplest mitochondria-related organelles (MROs) and possesses iron-sulfur (Fe-S) cluster synthesis (ISC) pathway. In this study, the authors identified bolA in an anaerobic protist, *G. intestinalis*, which was reported to be absent from anaerobic eukaryotes. They showed GibolA is localized in mitosomes and mitosomal GibolA is important for assembly of [4F-4S] cluster, which is essential for cytosolic enzyme, PFOR. Biochemical characterization of ISC pathway in highly degenerated MROs is important in mitochondrial evolutional study, and this study addressing this issue is potentially very interesting. However, their conclusions are sometimes too strong to be accepted as is. The specific points are detailing as follows;

**Major Point**

1. Line 29. The authors state, "specific interaction of IscA with the outer mitosomal membrane". I found IscA interactome data that suggests the interaction of IscA with MOMP35, but I could not find the data for the interaction with membrane.
2. MetalPredator is a publicly available software to predict iron-sulfur cluster binding proteins in protein sequence databases; therefore, as the authors state, "Of course, we cannot rule out the presence of a previously unknown protein with a unique cluster binding domain/motif in mitosomes.", it is highly possible to overlook. However, the author's conclusions regarding iron-sulfur cluster binding proteins are too strong; for example, line 300, "Given the apparent absence of the client proteins in the mitosomes". Line 334, "despite the loss of all mitochondrial pathways that require the presence of [4Fe-4S] clusters,"
3. Figure 1. It is OK as an introductory schematic figure, but the authors insert G. intestinalis enzymes information. However, the information included is so incomplete that readers may have difficulties to follow. It would be better to include authors' conclusions drawn in this study such as *Gi*BolA function, *Gi*IscA localization, and the assembly of cytosolic [4F-4S] clusters.
4. Figure 3A-C. How many times did authors repeat the cross-linking, protein isolation, and mass spectrometry analysis? Is the enrichment of MOMP35 in IscA sample statistically significant?
5. Line 523. The authors state, "each sample was done in triplicate". How variability in each sample is reflected in Volcano plots?
6. Line 166. Please include the data for "the analogous assay did not show any interaction between *Gi*BolA and *Gi*Nfu1" in supporting information.
7. The *bolA* gene knockout strain was able to be obtained and showed normal growth until 2 days, indicating that *bolA* gene is not essential. How does this strain acquire [4Fe-4S] for

PFOR?

**Minor Point**

1. Figure 1E. Which is GiBolA?
2. Line 306 and Materials and Methods. There is no description how the PFOR activity was assayed.
3. Figure 1D. Most readers will be misled abbreviations 'mGiGrx5' and 'mGiBolA' to 'mitosomal' or 'mitochondrial'. It would be easier for readers to abbreviate usual form, such as GiGrx5(C128A) (or GiGrx5$^{C128A}$) and GiBolA(H90A) (or GiBolA$^{H90A}$).
4. Line 136. Similar to 9, readers will confuse high-speed pellet (HSP) with heat-shock protein like Hsp70.
5. Line 382. The authors should consider removing (Grx??? Is this known?)
