# Supplementary material for: Adaptation of the late ISC pathway in the anaerobic mitochondrial organelles of Giardia intestinalis
Source: PLoS Pathog. 2023 Oct 4;19(10):e1010773. doi: 10.1371/journal.ppat.1010773 (PMC10578589; doi:10.1371/journal.ppat.1010773)
Supplement: Attachment — Submitted filename: response to the comments.docx [file ppat.1010773.s012.docx]

**Prague, 19 August 2023**

**Dear Editor, Reviewers,**

**Please find attached a revised version of our manuscript on the late ISC pathway in *Giardia intestinalis* mitosomes. It has been a year since the original submission. Why did it take so long? During this time, we have primarily been validating our strategy in developing and testing new cell lines lacking the BolA1 gene, as our attempts to reproduce some of our data in previously generated mutants failed. Specifically, it turned out that adding the gene back had no effect on PFOR activity in the cytosol. However, as it turned out, it was not the genetics of the cells that was the issue, but rather the highly variable PFOR activity between different experiments. Perhaps other factors such as oxidation, quality of the media and cell culture condition come into play in this case. To be sure, we generated several new delta-BolA1 cell lines and tested the effect on cell growth and cytosolic PFOR activity. However, none of the cell lines showed significant changes.**

**Therefore, we removed the PFOR and growth data from the entire manuscript. Reviewer 3 rightly asked about the reasons for the choice of this enzyme. It is the dominant metabolic enzyme in *Giardia* with Fe-S clusters and we expected a defect here.**

**However, there is obviously a long path from mitosomes to a [4Fe-4S] enzyme in the cytosol with many intermediate steps involved.**

**We then focused on whether these new delta-BolA1 cells instead have a problem with the assembly of [2Fe-2S] cluster, and using an in vitro assay we were able to show that cluster assembly remains unchanged. Similarly, no changes in iron uptake into cellular proteins could be detected in delta-BolA1 cells or in the newly established cell line lacking mitosomal Grx5. We thus toned down our general conclusion.**

**In addition, we changed the structure of manuscript, toned down and modified some of our strong expressions all over the manuscript. Below are our responses to all of reviewers comments.**

**Thanks a lot for looking after our manuscript.**

**Sincerely,**

**Pavel Dolezal**

Reviewer's Responses to Questions

Part I - Summary

Please use this section to discuss strengths/weaknesses of study, novelty/significance, general execution and scholarship.

Reviewer #1: Mitosome in G. intestinalis is one of the simplest mitochondria-related organelles (MROs) and possesses iron-sulfur (Fe-S) cluster synthesis (ISC) pathway. In this study, the authors identified bolA in an anaerobic protist, G. intestinalis, which was reported to be absent from anaerobic eukaryotes. They showed GibolA is localized in mitosomes and mitosomal GibolA is important for assembly of [4F-4S] cluster, which is essential for cytosolic enzyme, PFOR. Biochemical characterization of ISC pathway in highly degenerated MROs is important in mitochondrial evolutional study, and this study addressing this issue is potentially very interesting. However, their conclusions are sometimes too strong to be accepted as is.

Reviewer #2: Authors performed interactome analysis of the late iron sulfur cluster biosynthesis pathway in Giardia intestinalis mitosomes. It led to an interesting discovery of an aerobic mitochondrial protein homolog BolA involved in this mitosomal process. Microscopy and biochemical analyses indicated to BolA lozalization to Gi mitosomes. Validation of the interactome analysis by Y2H was performed satisfactorily. Authors also reported the apparent absence of a client protein for the synthesized [4Fe-4S] cluster in mitosomes. Instead, authors link this process to the cytosolic assembly of [4Fe-4S] clusters by knockout of the bolA gene. Validation of the interactome analysis by Y2H was performed. These findings contribute to the understanding of ISC synthesis in non-model organisms particularly among metamonads. Key questions although not currently answerable experimentally, were somehow addressed by speculation or hypotheses from the authors.

Reviewer #3: This short manuscript addresses the existence, and to some extent function, of the so-called late ISC proteins in Giardia intestinalis. Normally, mitosome-containing species like Giardia possess only the early part of the ISC machinery, and lack the late one because these species do not contain any known 4Fe-4S proteins, the targets of the late ISC system. The authors (re )identify the already characterized Grx5 and the genes for Isa2 and Nfu1 in the genome of Giardia. In a newly released genome assembly of Giardia they now also find Bol1, a protein which in mitochondrial FeS protein assembly at best has an auxiliary function. Ind1 and Iba57 (and presumably Isa1) was not found. The existence of so many Giardia late ISC proteins is surprising because to date no 4Fe-4S targets in Giardia mitosomes are known. The authors then concentrate mostly on Bol1 and show that a tagged overexpressed protein is located in membrane fractions of cell extracts, and localizes to particulate structures that are known to represent mitosomes. The protein interacts with many mitosomal proteins including the other late (and also early) ISC proteins. Vice versa, the tagged overexpressed late ISC proteins Nfu1, Isa2, and Grx5 interact with a similar set of proteins, as shown by affinity pulldowns. Interestingly, native Bol1 was not reliably identified in the mass spec data sets, and the authors suggest low abundance of this protein. Nevertheless, the proteomic analysis suggests an interaction of these late mitosomal ISC factors, but the functional issue is not further addressed in the current manuscript, simply because an obvious mitosomal 4Fe-4S target is missing. Also, the slightly different behavior of Isa2 in this analysis method was not further analyzed and thus remains descriptive (see point 7 below). The authors then generate a delta-bol1 cell using the now available CRISPR method for Giardia (unfortunately without confirming the specificity of the KO by complementation). The knockout cells grew slower, yet the number (and morphology? see point 9 below) of mitosomes remained unchanged. Finally, and a bit out of the blue, the authors check the activity of PFOR, a cytosolic 4Fe-4S protein. Why this one? Its activity is clearly down in Bol1 knockout cells, but a convincing explanation why this happened is not provided. Also, controls are missing. This effect could be, as claimed, a role of Bol1 for cytosolic FeS maturation, or (at this stage equally likely) a completely indirect consequence of Bol1 deletion. Overall, the interaction findings on Giardia Bol1 and the other late ISC proteins are nice and confirm findings in many other organisms. Functionally, the data is quite preliminary and puzzling at this stage. Would knockouts of the other late ISC factors give the same result for PFOR? Is the effect due to the Grx5-Bol1 complex helping Fdx1 maturation and hence causing an early ISC defect? Many questions are unanswered that are central for the claim of a crosstalk between mitosomes and cytosol.

**Thanks for the careful review. As state above, in the revised version of the manuscript, we removed the impact of mitosomal ISC pathway on the activity of cytosolic PFOR and the cell growth. We added new in vitro assay [2Fe-2S] assembly data and iron incorporation experiment to characterize the role of BolA1 and partly Grx5 in the mitosomal ISC pathway. We toned down our strong expressions and conclusions.**

**Response to general comments**

Part II – Major Issues: Key Experiments Required for Acceptance

Please use this section to detail the key new experiments or modifications of existing experiments that should be absolutely required to validate study conclusions.

Reviewer #1: Major Point

1. Line 29. The authors state, “specific interaction of IscA with the outer mitosomal membrane”. I found IscA interactome data that suggests the interaction of IscA with MOMP35, but I could not find the data for the interaction with membrane.

**This was innacurate expressions, there should be „…interaction with the outer mitosomal membrane proteins“ it was corrected.**

2. MetalPredator is a publicly available software to predict iron-sulfur cluster binding proteins in protein sequence databases; therefore, as the authors state, “Of course, we cannot rule out the presence of a previously unknown protein with a unique cluster binding domain/motif in mitosomes.”, it is highly possible to overlook. However, the author’s conclusions regarding iron-sulfur cluster binding proteins are too strong; for example, line 300, “Given the apparent absence of the client proteins in the mitosomes”. Line 334, “despite the loss of all mitochondrial pathways that require the presence of [4Fe-4S] clusters,”

**Thanks, yes, we are aware of the holes in this approach. We have toned down the expressions, but for mitochondrial pathways that rely on [4Fe-4S] proteins, we can be almost sure that no such protein remains in *Giardia,* as these proteins are absent in other metamonad species and perhaps the entire supergroup. But of course, an absence cannot be proved.**

3. Figure 1. It is OK as an introductory schematic figure, but the authors insert G. intestinalis enzymes information. However, the information included is so incomplete that readers may have difficulties to follow. It would be better to include authors’ conclusions drawn in this study such as GiBolA function, GiIscA localization, and the assembly of cytosolic [4F-4S] clusters.

**We have included background description of the pathway in the figure legend.**

4. Figure 3A-C. How many times did authors repeat the cross-linking, protein isolation, and mass spectrometry analysis? Is the enrichment of MOMP35 in IscA sample statistically significant?

**All pulldowns were performed in biological and triplicates, and only statistically significant data are shown in the volcano plot and used for later analyses.**

5. Line 523. The authors state, “each sample was done in triplicate”. How variability in each sample is reflected in Volcano plots?

**The variability between samples is represented by significance of each protein in the volcano plot as the value was calculated from three independent pulldowns. This value was then plotted against the enrichment obtained from these three experiments. Only proteins which appeared in all three experiments are shown. The enrichments/fold changes are shown with their average values.**

6. Line 166. Please include the data for “the analogous assay did not show any interaction between GiBolA and GiNfu1” in supporting information.

**The experimental data were added**

7. The bolA gene knockout strain was able to be obtained and showed normal growth until 2 days, indicating that bolA gene is not essential. How does this strain acquire [4Fe-4S] for PFOR?

**After generating three new clones of bolA gene knockout, we removed this data from the paper as not reproducible.**

Reviewer #2: (No Response)

Reviewer #3: Other comments:

1. Lanes 45,46: This half sentence sounds as if mitochondria have more than one pathway for FeS synthesis. What the authors mean is that there are other pathways in addition to the FeS. Please rephrase.

**It was corrected**

2. Lanes 52 onwards: It is confusing that the authors use the bacterial nomenclature for most ISC proteins. For instance, IscS in fact is Nfs1 or NFS1. Calling the mitochondrial-mitosomal proteins IscS etc. is incorrect. I therefore recommended using either the yeast or human names for these proteins.

**Thanks for this comment. We are aware that the nomenclature is confusing and to some extent unfortunate. We prefer to keep the name of the genes as they have been historically named in *Giardia*, the object of the study.**

3. Lanes 90-92: The statement “that mitosomal BolA, and thus the late ISC pathway, is required for the formation of cytosolic 4Fe-4S clusters.” is incorrect in that the functionality of the late ISC pathway was not touched upon in this work, just Bol1 was looked at.

**Yes, the sentence was removed in the new version of the manuscript.**

4. Lane 98 ff.: Define in Suppl. Fig. 1B that it is NOT Isa1 (ISCA1) but Isa2 (ISCA2) in the alignment. The latter proteins from yeast and humans are used. Hence, use IscA2 rather than IscA (or even better, the name as recommended in point 2).

**Name of GiIscA was changed to GiIscA2 all over the manuscript. Also BolA was labelled as BolA1 to reflect its closer relationship to its BolA1 orthologues.**

5. Fig. 2F is unclear to me. What do the authors mean by “absent from the transcriptome”? Gene present, but not transcribed?

**The term means that no genomic data are available and only transcriptomic data were used. This information was also added to the legend.**

6. Lane 131: The headline “GiBolA is part of mitosomal late ISC pathway” is an exaggeration. What was described in the respective chapter is the interaction of Bol1 with Giardia proteins, mostly located in mitosomes, not a pathway. The authors may therefore wanna be a bit more accurate in phrasing the headline.

**The headline was corrected accordingly.**

7. Lanes 257-258: This statement is a bit of an exaggeration at this stage. This behavior may simply be a different membrane sticking of Isu1 and Isa2 proteins. Without further proof (e.g., salt variations; affinity purifications of Isu1, etc.) I suggest to tone down the statement.

**We toned down the statement and included different physical properties of the proteins as another explanation of the observed behavior.**

8. Lanes 289-291: Rephrase the sentence, please. It seems incomplete (or the two parts are not connected properly?).

**Thanks, the sentence was corrected.**

9. Lane 298: Typo: Suppl. Fig. 3 instead of 2. More important: How did the authors define “morphology”? Simply by an unchanged particulate appearance in IF? If yes, this should be stated explicitly, because it would not really reflect morphology (which in my view would need EM analysis).

**Thanks, we corrected the number of the figure and we changed the statement about the mitosomal morphology to just the number of the organelles. The EM analysis of the mitosomes is still a challenging technique.**

Part III – Minor Issues: Editorial and Data Presentation Modifications

Please use this section for editorial suggestions as well as relatively minor modifications of existing data that would enhance clarity.

Reviewer #1: Minor Point

1. Figure 1E. Which is GiBolA?

**We added the label to the tree.**

2. Line 306 and Materials and Methods. There is no description how the PFOR activity was assayed.

**It was a mistake but we have removed PFOR assay from the revised t version of the manuscript**

3. Figure 1D. Most readers will be misled abbreviations ‘mGiGrx5’ and ‘mGiBolA’ to ‘mitosomal’ or ‘mitochondrial’. It would be easier for readers to abbreviate usual form, such as GiGrx5(C128A) (or GiGrx5C128A) and GiBolA(H90A) (or GiBolAH90A).

**Thanks, the labelling was changed accordingly.**

4. Line 136. Similar to 9, readers will confuse high-speed pellet (HSP) with heat-shock protein like Hsp70.

**Thanks, we understand the confusion it may create but we left HSP abbreviations in place as it is somewhat historically used in giardia field. There is explanation of the abbreviated term in the text.**

5. Line 382. The authors should consider removing (Grx??? Is this known?)

**We removed that as it was a mistake ☺**

Reviewer #2: Please describe the mitosomal marker GL50803_9296 in line 135.

**We added a reference to this protein/antibody. It is a protein of unknown function but the specific polyclonal antibody raised against the protein gives the best results for the immunofluorescent labelling.**

Edit line 382.

Review the entire text for scientific names are not italicized.

**Done**

Reviewer #3: (No Response)
